# Comparative Study of the Effect of 5,6-Dihydroergosterol and 3-*epi*-5,6-dihydroergosterol on Chemokine Expression in Human Keratinocytes

**DOI:** 10.3390/molecules25030522

**Published:** 2020-01-25

**Authors:** Ye Seul Park, Hye Jin Moon, Kwang Hyun Ahn, Tae Hoon Lee, Hakwon Kim

**Affiliations:** Department of Applied Chemistry and Global Center for Pharmaceutical Ingredient Materials, Kyung Hee Uinveristy, Yongin-si, Gyeonggi-do 17104, Korea; pys3981@naver.com (Y.S.P.); hjm6719@naver.com (H.J.M.); khahn@khu.ac.kr (K.H.A.)

**Keywords:** anti-inflammation, CCL17/TARC, CCL22/MDC, 3-*epi*-5,6-dihydroergosterol glycoside, chronic dermatitis treatment

## Abstract

5,6-Dihydroergosterol-glucose is an organic synthetic derivative of spinasterol-glucose, which has potent anti-inflammatory activity. We previously synthesized alpha and beta anomers of DHE-glycosides and compared their inhibitory activity on CCL17 and CCL22 mRNA expression induced by TNF-α/IFN-γ in activated HaCaTs. Recently, we synthesized a new type of DHE-glycosides, 3-*epi*-5,6-dihydroergosterol(3-*epi*-DHE)-glycosides, and compared its inhibitory activity on mRNA expression levels of CCL17 and CCL22 in TNF-α/IFN-γ-induced HaCaT. DHE-Xly did not affect TNF-α/IFN-γ induced CCL17 and CCL22 mRNA expression in HaCaTs, however, 3-*epi*-DHE-Xly strongly inhibited TNF-α/IFN-γ induced CCL17 and CCL22 mRNA expression levels in human keratinocytes. These results provide important clues for development of chronic dermatitis treatment via inhibition of chemokine expression using DHE derivatives.

## 1. Introduction

In general, structural changes according to epimerization in several synthetic compounds are known to present different biological activities. In the case of vitamin D, the epimeric compound has traditionally been reported to be inactive, but a recent study has shown that C3-*epi* 25 (OH) D3 affects growth and bone mineral density. It has also been reported that C3-*epi* 25 (OH) D3 has distinctly different biological activities and that further studies are needed for verification [1,2]. 

5,6-Dihydroergosterol-glucose (DHE-Glc) is an organic synthetic derivative of spinasterol-glucose, which is a phytosterol isolated from the *Srewarita Koreana* leaf extract [3]. In several studies, spinasterol-Glc was found to have various physiological activities as well as potent anti-inflammatory activity, and our previous study confirmed that its derivative DHE-Glc also had various biological activities [4,5]. In particular, it was confirmed that DHE-Glc strongly inhibits the mRNA and protein expression levels of pro-inflammatory cytokines and chemokines in human keratinocytes (HaCaTs) activated by TNF-α/IFN-γ. In addition, DHE-Glc was found to have potent anti-inflammatory activity in the 2,4-dinitrochlorobenzene (DNCB)-induced chronic dermatitis mouse animal model [5].

Chemokines are cytokines with chemotaxis and cytokine or signal transduction proteins secreted from various cell types including endothelial cells, epithelial cells, and immune cells after stimulation by external and/or internal stimuli [6]. Chemokines are classified into four main subfamilies—CXC, CC, CX3C, and XC—depending on the important four cysteine residues in the conserved position. These proteins are known to exert biological activities by interacting with specific binding to G protein-linked coupled receptors (GPCRs), which are selectively found on the surface of each of their target cells [7]. In several recent studies, thymus- and activation-regulated chemokine (TARC/CCL17) and monocyte-derived chemokine (MDC/CCL22) belonging to CC-chemokine are reported as important biomarkers in chronic dermatitis [8,9]. It has been reported that levels of CCL17 and CCL22 are increased in the blood and dermatitis skin tissues of many chronic dermatitis patients, causing continuous immune cell activation [10]. 

We previously have reported that alpha and beta anomers of DHE-glycosides are produced during the synthetic process of DHE-glycoside compounds. Also, we compared the inhibitory activity of these compounds on CCL17 and CCL22 mRNA expression in HaCaTs activated by TNF-α/IFN-γ and it confirmed that the beta anomer of DHE glycoside has a greater effect than the alpha anomer of DHE-glycoside on the expression levels of CCL17 and CCL22 in TNF-α/IFN-γ -induced HaCaT cells. This result was confirmed to be probably due to the stability of both compounds [4]. In this study, we synthesized a new type of DHE-glycosides, such as 3-*epi*-5,6-dihydroergosterol(3-*epi*-DHE)-glycosides, and compared the inhibitory activity of mRNA expression levels of CCL17 and CCL22 in TNF-α/IFN-γ-induced HaCaT. These results provide important clues for development of chronic dermatitis treatment via inhibition of chemokine expression using DHE derivatives. 

## 2. Results and Discussion

**Scheme 1:**
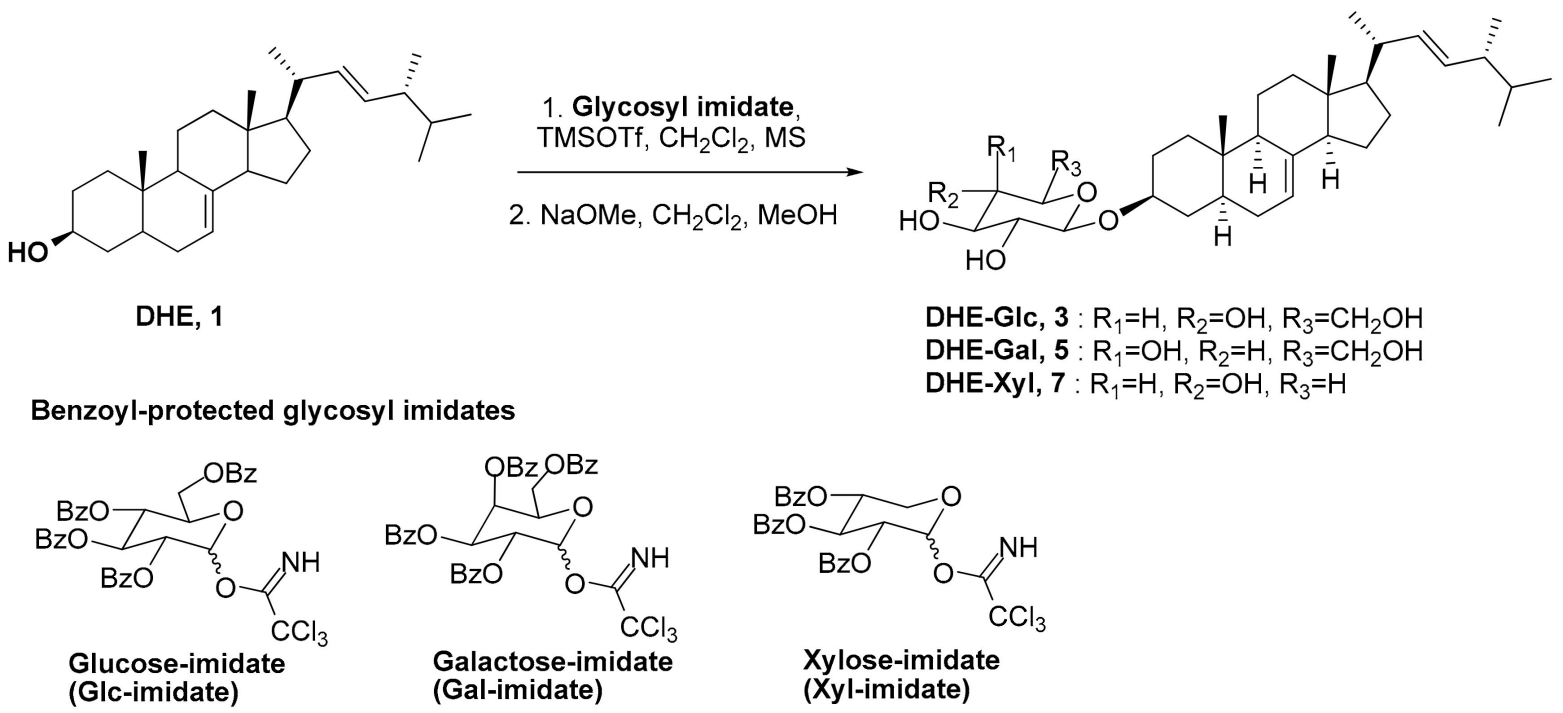
Synthesis of DHE glycosides by Schmidt glycosylation.

In previous studies, 5,6-dihydroergosterol (**1**, DHE, Stellarsterol) was selected as a new steroid with the same steroid backbone as spinasterol but with a different side chain [3]. In a known synthetic method, DHE **1** has been synthesized from ergosterol using DIBAL-H reduction [3,11] and benzoyl-protected glycosyl trichloroacetimidates (Gluccose-imidate, Galactose-imidate, and Xylose-imidate) were synthesized from glucose, galactose and xylose, respectively, via benzoyl protection, bromination, hydroxylation, and trichloroacetimidation [11]. We have already reported that β-anomers of DHE glycosides (DHE-Glc **3**, DHE-Gal **5** and DHE-Xyl **7**) have been synthesized by Schmidt glycosylation of DHE with glycosyl imidates, as shown in Scheme 1, and exhibit anti-inflammatory activity [3,4].

**Scheme 2:**
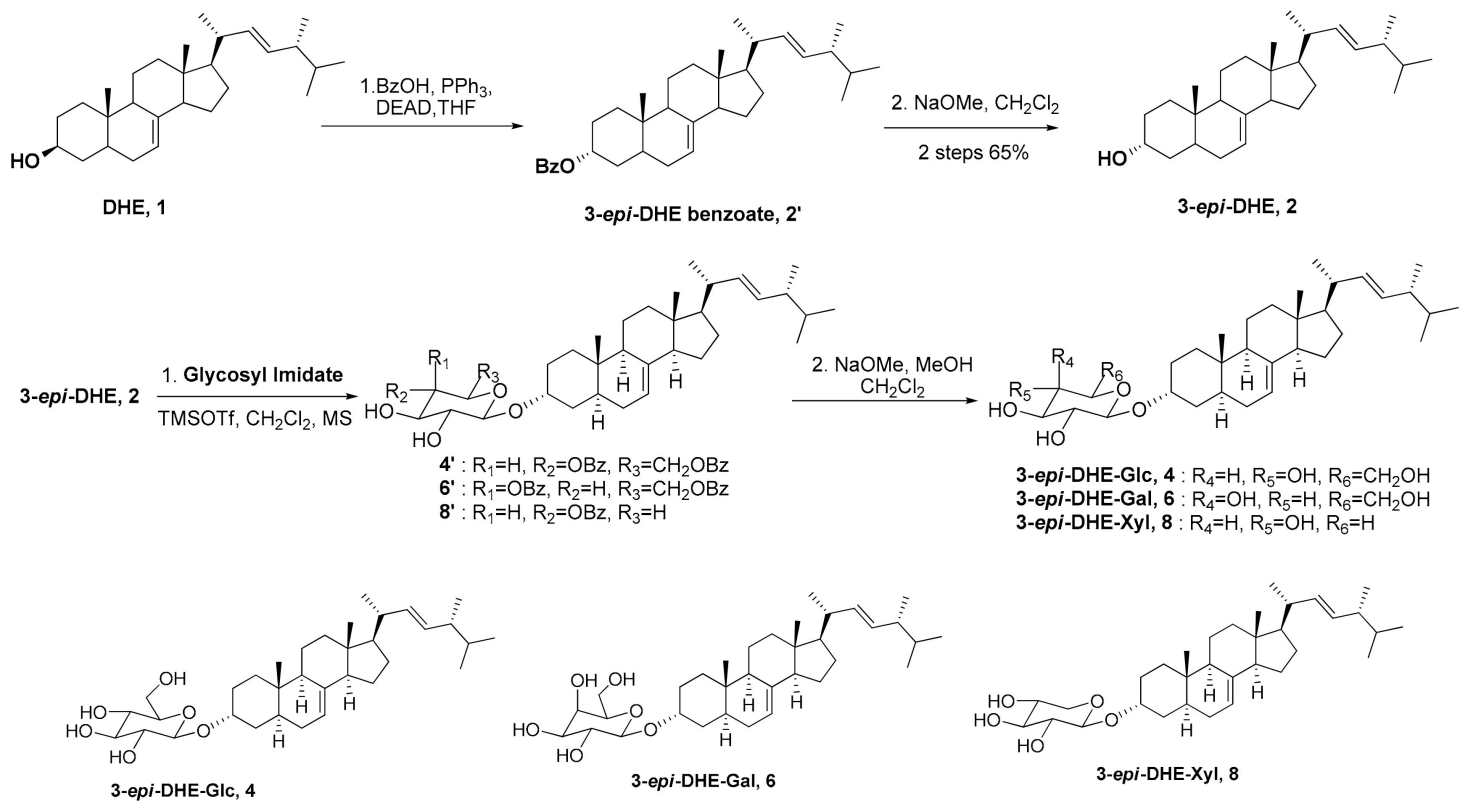
Synthesis of 3-*epi*-DHE **2** via Mistunobu reaction and its glycosides by Schmidt glycosylation.

We were curious how 3-*epi*-DHE-glycosides (**4**, **6**, and **8**), which differ in the stereochemistry of the hydroxyl group at the C-3 position of DHE **1**, differ from previous DHE-glycosides (**3**, **5**, and **7**) in terms of bioactivity. Therefore, in order to compare the physiological activity of two epimers, synthesis of 3-*epi*-DHE **2** was required, first. 3-*epi*-DHE **2** was thought to be synthesized by Mitsunobu reaction of DHE **1**. Mitsunobu reaction of DHE **1** using benzoic acid, DEAD, and triphenylphosphine produced 3-*epi*-DHE **2** in a good yield [12,13]. Its structure was confirmed by comparing the peak at C-3 of DHE **1** (δ 3.06) with the C-3 peak of 3-*epi*-DHE **2** (δ 4.07) on ^1^H-NMR, where the proton peak at C-3 of 3-*epi*-DHE **2** shifted downfield. The *epi*-DHE glycoside synthesis proceeded in the same way as DHE glycoside synthesis. As a result, three 3-*epi*-DHE-glycosdies, such as 3-*epi*-DHE-glucose (3-*epi*-DHE-Glu, **4**), 3-*epi*-DHE-Galactose (3-*epi*-DHE-Gal, **6**), and 3-*epi*-DHE xylose (3-*epi*-DHE-Xyl, **8**), were obtained [Scheme 2]. 

We examined the effects of the *epi*-DHE-glycoside on CCL17 and CCL22 mRNA expression in TNF-α/IFN-γ-induced HaCaT cells. The cell cytotoxicity of HaCaT cells treated with 3-*epi*-DHE-glycoside (**4**, **6**, and **8**) was determined with MTT assays (data not shown). The cell viability of the other synthetic DHE glycoside compounds did not have a cytotoxic effect up to 20 μM in our previous studies [3,4].

**Figure 1:**
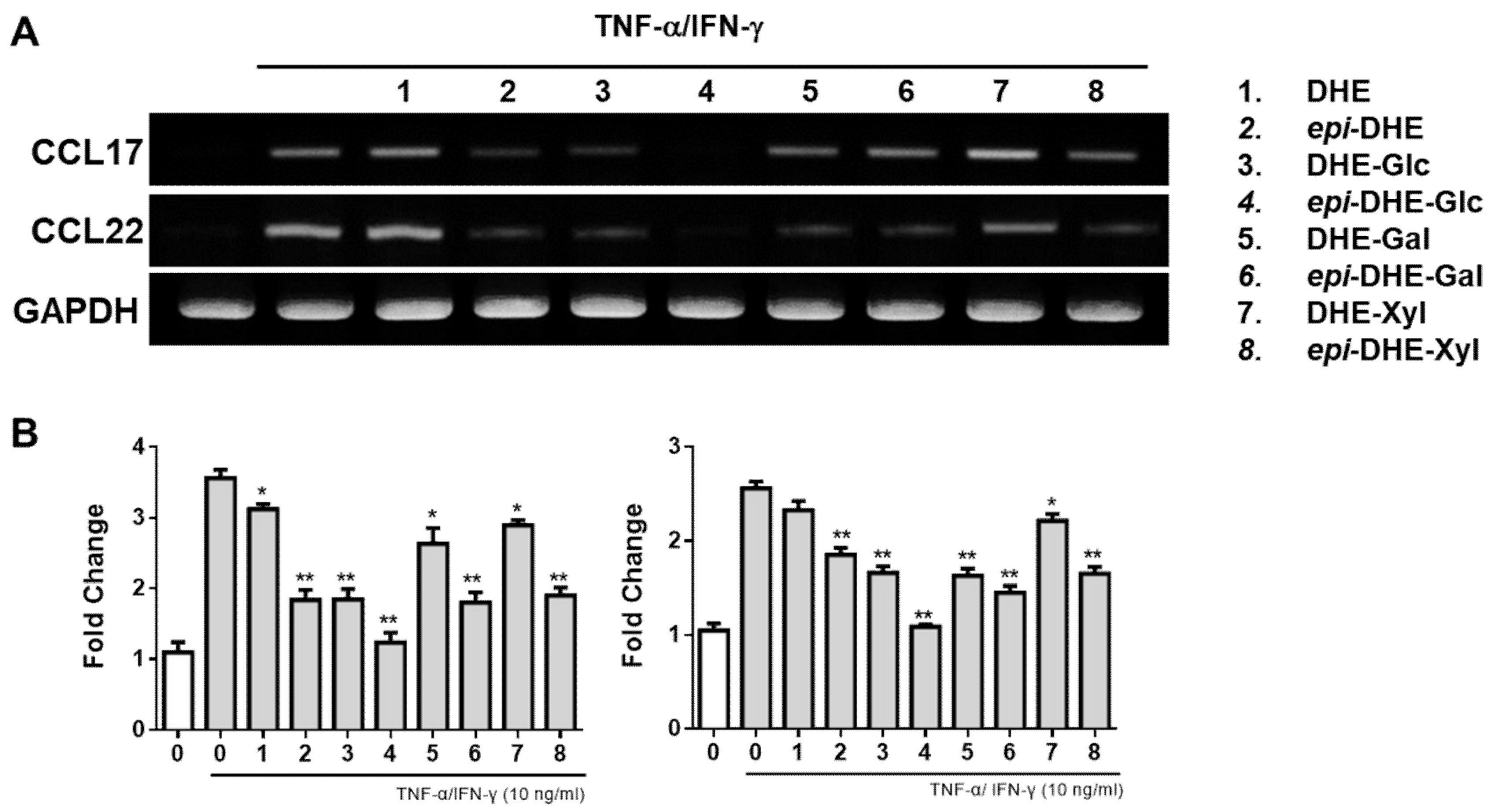
Effects of epimers of DHE on CCL17 and CCL22 mRNA expression. CCL17 and CCL22 mRNA levels were determined using an RT-PCR assay (**A**). mRNA expression levels of the chemokines CCL17 (left) and CCL22 (right) were normalized to GAPDH by quantitative real-time-PCR (**B**). All experiments were performed in triplicate and repeated at least three times. * *p* < 0.05 compared to treatment with positive control, ** *p* < 0.01 compared to treatment with positive control.

**Figure 2:**
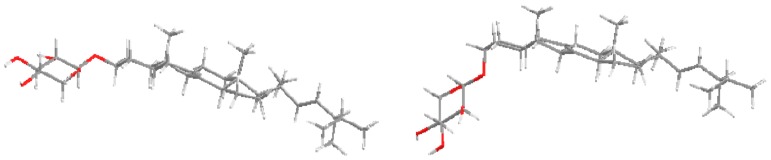
Graphical representation of structures of DHE-xylose 7(left) and 3-*epi*-DHE-xylose 8(right) by DFT calculation. Grey (carbon), white (hydrogen), and red (oxygen) bond.

We tested the mRNA expression levels of CCL17 and CCL22 using an RT-PCR assay in TNF-α/IFN-γ-treated HaCaT cells. Furthermore, we confirmed mRNA expression levels of CCL17 and CCL22 by real-time PCR assay (Figure 1). Analysis of the mRNA expression of CCL17 and CCL22, it was confirmed that the positive control group treated with TNF-Α/IFN-Γ (10 ng/mL) increased about 3-to 4-folds compared to that of the negative control group treated with media only. Examining the mRNA expression of CCL17, it was confirmed that only the DHE-Glc **3** (20 μM) had a strong inhibitory activity, as in the previous results [3,4]. On the other hand, the 3-*epi*-DHE glycosides including 3-*epi*-DHE **2**, 3-*epi*-DHE-Glc **4**, 3-*epi*-DHE-Gal **6**, and 3-*epi*-DHE-Xyl **8** increased the activity in all compounds (20 μM). As shown in quantitative PCR (Figure 1B), we could confirm that the change of structure of DHE-Xyl **7** on the DHE-glycoside affects both CCL17 and CCL22 mRNA expression levels. DHE-Xly **7** did not affect on TNF-α/IFN-γ-induced CCL17 and CCL22 mRNA expression in HaCaTs, however, 3-*epi*-DHE-Xly **8** inhibited TNF-α/IFN-γ induced CCL17 and CCL22 mRNA expression levels in human keratinocyte. Prediction of the stable structure of DHE-glycoside and its epimer, 3-*epi*-DHE-glycoside, by DFT calculation showed obvious differences. As shown in Figure 2, DHE-xylose **7** has a relatively linear shape, while 3-*epi*-DHE-xylose **8** has a curved shape. In addition, the calculated energy difference between the two epimers is only 0.70 kcal/mol. Therefore, the structural difference between the two epimers likely influenced the change of bioactivity.

## 3. Materials and Methods 

### 3.1. Synthesis of 3-epi-5,6-dihydroergosterol (3-epi-DHE, ***2***)

3-e*pi*-Benzoate-5,6-dihydroergosterol (2′): 5,6-Dihydroergosterol (0.1 g, 0.251 mmol) in dry THF (4.2 mL) was added to benzoic acid (0.074 g, 0.602 mmol) and triphenylphosphine (0.158 g, 0.602 mmol). After slow addition of DIAD (0.128 mL, 0.653 mmol) at 0 °C, the mixture was stirred at room temperature overnight. The mixture was concentrated in vacuo and purified by flash column chromatography (ethyl acetate/hexane) to produce a white solid. Yield: 74%. m.p. 112–124 °C. ^1^H-NMR (300 MHz, CDCl_3_) δ 0.54–2.08 (m, 41H, peaks from steroidal structure), 5.12–5.28 (m, 3H), 5.31 (br s, 1H), 7.44 (t, 2H, *J* = 7.5 Hz), 7.55 (t, 1H, *J* = 7.3 Hz), 8.05 (d, 2H, *J* = 7.2 Hz).

3-e*pi*-5,6-Dihydroergosterol (**2**): 3-*epi*-Benzoate-5,6-dihydroergosterol **2′** (0.094 g, 0.186 mmol) in methylene chloride (9.31 mL) was added to NaOMe in MeOH (4.37 M, 0.21 mL, 0.931 mmol). The mixture was stirred at room temperature overnight. After neutralization by Dowex Mac-3, the mixture was filtered. The filtrate was concentrated in vacuo and purified by flash column chromatography (ethyl acetate/hexane) to produce a white solid. Yield: 88%. m.p. 188–198 °C. ^1^H-NMR (300 MHz, CDCl_3_) δ 0.55 (s, 3H), 0.78 (s, 3H), 0.76–0.80 (m, 6H), 0.91 (d, 3H, *J =* 6.8 Hz), 0.93 (d, 3H, *J =* 6.6 Hz), 1.17–2.06 (m, 23H, peaks from steroidal structure), 4.07 (br. s., 1 H), 5.08–5.29 (m, 3 H). ^13^C-NMR (75 MHz, CDCl_3_) δ (ppm): 12.1, 17.7, 19.7, 20.00, 21.2, 21.3, 22.9, 28.2, 28.8, 29.6, 32.0, 33.2, 34.7, 34.8, 35.6, 39.5, 40.6, 42.9, 43.4, 49.5, 55.2, 56.0, 66.6, 117.7, 132.0, 135.9, 139.7.

### 3.2. General Synthetic Procedures of Glycosylation and Hydrolysis

#### 3.2.1. Synthesis of (3α, 22E)-ergosta-7,22-dien-3-yl-β-d-glucopyranoside (3-*epi*-DHE-β-Glc, **4**) 

2,3,4,6-Tetrabenzoyl-β-glucopyranosyl 3-*epi*-dihydroergosterol (**4**′): 2,3,4,6-Tetra-*O*-benzoyl-glucopyranosyl trichloroacetimidate (0.335 g, 0.452 mmol), 3-*epi*-DHE **2** (0.09 g, 0.226 mmol), and a 4Å molecular sieve in methylene chloride (5.6 mL) were stirred at 0 °C. After addition of TMSOTf (0.0086 mL, 0.045 mmol) at 0 °C, the mixture was stirred at room temperature for 2 hr. After neutralization by Et_3_N, the mixture was filtered and concentrated in vacuo and purified by flash column chromatography (ethyl acetate/hexane) to produce a white solid. Yield: 61%. m.p. 96–98 °C. ^1^H-NMR (300 MHz, CDCl_3_) δ 0.50–2.29 (m, 42H, peaks from steroidal structure), 3.98–4.08 (m, 1H), 4.09–4.19 (m, 1H), 4.44–4.55 (m, 1H), 4.58–4.68 (m, 1H), 4.76–5.02 (m, 1H), 5.15–5.30 (m, 2H), 5.50–5.63 (m, 1H), 5.63–5.74 (m, 1H), 5.58–5.99 (m, 1H), 7.28–7.55 (m, 12H), 7.82–8.03 (m, 8H).

3-*Epi*-DHE-β-Glc, **4**; 2,3,4,6-Tetrabenzoyl-β-glucopyranosyl 3-*epi*-dihydroergosterol **4**′ (0.13 g, 0.133 mmol) in a mixed solvent (CH_2_Cl_2_:MeOH = 6.7 mL:6.7 mL) was added to NaOMe in MeOH (4.37 M, 0.183 mL, 0.798 mmol). The mixture was stirred at room temperature overnight. After neutralization by Dowex Mac-3, the mixture was filtered. The filtrate was concentrated in vacuo and purified by flash column chromatography (CH_2_Cl_2_/MeOH) to produce a white solid. Yield: 76%. m.p. 144.9–151.1 °C. ^1^H-NMR (300 MHz, Pyridine-D_5_) δ 0.70 (s, 2H), 0.75 (s, 1H), 0.89 (dt, 6H, *J* = 6.4, 3.0 Hz), 0.91–0.96 (m, 3H), 0.96–1.01 (m, 3H), 1.09 (d, 3H, *J* = 6.6 Hz), 1.11–2.34 (m, 23H, peaks from steroidal structure).3.94–4.03 (m, 1H), 4.05–4.16 (m, 1H), 4.26–4.37 (m, 3H), 4.40–4.51 (m, 1H), 4.54–4.64 (m, 1H), 4.89–4.96 (m, 2H), 5.25–5.36 (m, 2H). ^13^C-NMR (75 MHz, Pyridine-D_5_) δ 11.20, 12.04, 17.17, 17.86, 18.64, 19.83, 20.15, 21.30, 20.53, 25.52, 33.32, 35.03, 35.16, 36.10, 37.45, 39.29, 39.59, 42.76, 43.05, 43.09, 47.00, 49.36, 57.15, 62.84, 71.71, 72.88, 75.37, 78.40, 78.71, 102.29, 102.53, 127.24, 132.07, 142.14. IR; OH peak at 3068–3602 cm^−1^.

#### 3.2.2. Synthesis of (3α, 22E)-ergosta-7,22-dien-3-yl-β-d-galactopyranoside (3-*epi*-DHE-β-Gal, **6**)

2,3,4,6-Tetrabenzoyl-β-galactopyranosyl 3-epi-dihydroergosterol (**6′**); Yield: 79%. m.p. 100–103 °C. ^1^H-NMR (300 MHz, CDCl_3_) δ 0.50–2.35 (42H, peaks from steroidal structure), 3.56–3.75 (m, 1 H), 4.27–4.38 (m, 1 H), 4.40–4.47 (m, 1H), 4.66–4.72 (m, 1H), 4.93 (d, 1H, J = 7.7 Hz), 5.10–5.25 (m, 2H), 5.60 (dd, 1H, J = 3.2, 10.4 Hz), 5.75–5.81 (m, 1H), 5.99 (d, 1H, J = 2.8 Hz), 7.21–7.26 (m, 2H), 7.35–7.63 (m, 10H), 7.80 (d, 2H, J = 7.5 Hz), 7.96 (d, 2H, J = 7.5 Hz), 8.03 (d, 2H, J = 8.1 Hz), 8.11 (d, 2H, J = 7.3 Hz).

3-*epi*-DHE-β-Gal, **6**; Yield: 93%. m.p. 139.5–146.5 °C. ^1^H-NMR (300 MHz, Pyridine-D_5_) δ 0.69–0.74 (m, 3H), 0.84—0.93 (m, 6 H), 0.94–1.00 (m, 3H), 1.02–1.16 (m, 6H), 1.26–2.39 (m, 23H, peaks from steroidal structure) 4.10–4.12 (m, 1H), 4.21–4.24 (m, 1 H), 4.32 (s, 1H), 4.46–4.56 (m, 3 H), 4.64 (s, 1H), 4.86 (t, 1H, *J* = 9 Hz), 5.07–5.22 (m, 1H), 5.27–5.45 (m, 2H). ^13^C-NMR (75MHz, Pyridine-D_5_) δ 11.23, 12.07, 17.89, 18.67, 19.83, 19.86, 20.18, 21.57, 29.25, 30.00, 33.36, 35.06, 36.08, 36.24, 37.44, 39.25, 39.62, 42.77, 43.08, 43.13, 47.02, 49.33, 57.16, 62.42, 70.30, 72.69, 75.44, 75.48, 76.85, 103.28, 127.29, 132.09, 136.17 142.10. IR; OH peak at 3008–3640 cm^−1^).

#### 3.2.3. Synthesis of (3α, 22E)-ergosta-7,22-dien-3-yl-β-d-xylopyranoside (3-*epi*-DHE-β-Xyl, **8**)

2,3,4 -Tribenzoyl-β-xylopyranosyl 3-*epi*-dihydroergosterol (**8′**)**;** Yield: 66%. m.p. 100–103 °C. ^1^H-NMR (300 MHz, CDCl_3_) δ 0.57–2.37 (m, 42H, peaks from steroidal structure), 3.66 (dd, 1H, *J* = 11.9, 8.1 Hz), 4.03 (br. s., 1H), 4.44 (dd, 1H, *J* = 11.9, 4.6 Hz), 4.84–4.91 (m, 1H), 5.17–5.27 (m, 2H), 5.32 (tt, 1H, *J* = 7.7, 4.7 Hz), 5.38–5.49 (m, 1H), 5.80 (q, 1H, *J* = 8.0 Hz), 7.31–7.43 (m, 6H), 7.46–7.59 (m, 3H), 8.00 (dt, 6H, *J* = 4.5, 3.1 Hz). 

3-*epi*-DHE-β-Xyl, **8**; Yield: 82%. m.p. 180–186 °C. ^1^H-NMR (300 MHz, Pyridine-D_5_) δ 0.70–0.78 (m, 3H), 0.86–0.91 (m, 6H), 0.92–1.01 (m, 6H), 1.07–1.11 (d, 3H, J = 6.6 Hz), 1.12–2.40 (m, 23H, peaks from steroidal structure), 3.70–3.81 (m, 1H), 4.02–4.15 (m, 1H), 4.17–4.37 (m, 3H), 4.41 (dd, 1H, J = 11.0, 5.1 Hz), 4.79–4.89 (m, 1H), 5.06 (m, 1H), 5.26–5.35 (m, 2H). ^13^C-NMR (75MHz, Pyridine-D_5_) δ 11.28, 12.13, 17.24, 17.90, 17.95, 18.71, 19.90, 20.22, 21.38, 21.62, 26.08, 30.08, 33.42, 35.19, 36.22, 37.58, 39.70, 42.87, 43.16, 43.21, 47.12, 49.54, 57.26, 67.41, 71.43, 73.49, 75.33, 78.82, 103.79, 127.47, 132.32, 136.40, 142.44. IR; OH peak at 3029–3594 cm^−1^.

### 3.3. Cell Culture

Human keratinocytes (HaCaT) were cultured as follows. We used Dulbecco’s modified Eagles medium (Welgene, Seoul, Korea) containing 10% fetal bovine serum (FBS), 100 units/mL penicillin, and 100 μg/mL streptomycin (Gibco BRL, Rockville, MD). Cells were cultured in an incubator at 95% humidity and 5% (*v*/*v*) air/CO_2,_ at 37 °C.

### 3.4. Reverse Transcriptase-Polymerase Chain Reaction (RT-PCR) Analysis

Total RNA was isolated from samples of treated HaCaTs using BioZol reagent (Bio-Solution, Seoul, Korea) according to the manufacturer’s recommendations. cDNA was established using reverse-transcriptase M-MuLV (Fermentas Life Science, Pittsburgh, PA, USA). PCR amplification was confirmed using specific primers as follows (Bioneer, Daejeon, Korea): CCL17 (forward) 5′-ACT GCT CCA GGG ATG CCA TCG TTT TT-3′, (reverse) 5′-ACA AGG GGA TGG GAT CTC CCT CACTG-3′, CCL22 (forward) 5′-AGG ACA GAG CAT GGC TCG CCT ACA GA-3′, and (reverse) 5′- TAA TGG CAG GGA GGT AGG GCT CCT GA-3′. GAPDH mRNA was used as an internal control.

### 3.5. Quantitative Real-Time PCR

RNA samples and cDNA were obtained using the same methods as for RT-PCR analysis. Reverse transcription reaction was carried out by using AccuPower RT Premix (Bioneer, Daejeon, Korea) according to the manufacturer’s recommendations. Real-time PCR was conducted in a Lightcycler Nano system (Roche, Mannheim, Germany) using LightCycler DNA Master SYBER GREEN I (Roche, Mannheim, Germany). The mRNA expression levels were normalized to GAPDH by calculating delta Ct values. The specific primers as follows: CCL17 (forward) 5′-AGG GAC CTG CAC ACA GAG AC-3′, (reverse) 5′-CTC GAG CTG CGT GGA TGT GC-3′; CCL22 (forward) 5′-ATG GCT CGC CTA CAG ACT GCA CTC-3′ (reverse) 5′-CAC GGC AGC AGA CGC TGT CTT CCA-3′.

### 3.6. Statistical Analysis

Unless otherwise stated, all experiments were performed with triplicate samples and repeated at least three times. Data are presented as means ± S.D. and statistical comparisons between groups were performed using one-way ANOVA followed by a Student’s *t-*test.

## 4. Conclusions

In conclusion, we synthesized 3-*epi*-5,6-dihydroergosterol (**2**) with reverse stereochemistry of the C3 position in DHE **1** using the Mitsunobu reaction. The compound was compared with previously synthesized DHE and its glycosides in terms of anti-inflammatory effects. The 3-*epi*-DHE glycosides, 3-*epi*-DHE-Glc **4**, 3-*epi*-DHE-Gal **6** and 3-*epi*-DHE-Xyl **8** showed better activity than the DHE glycosides (**3, 5** and **7**). In particular, among epimeric DHE glycosides, 3-*epi*-DHE-Xyl **8** showed significantly better activity than DHE-Xyl **7**.
